# Individualised N-terminal Pro-B-type Natriuretic Peptide Thresholds for the Diagnosis of Heart Failure: A Proof-of-Concept Study

**DOI:** 10.7759/cureus.108732

**Published:** 2026-05-12

**Authors:** Nicholas Black, Fardad Soltani, Joshua Bradley, Simon G Williams, Anna B Reid, Gaetano Nucifora, Matthias Schmitt, Mark C Petrie, Christopher A Miller

**Affiliations:** 1 Division of Cardiovascular Sciences, University of Manchester, Manchester, GBR; 2 Department of Cardiology, Manchester University NHS Foundation Trust, Manchester, GBR; 3 School of Cardiovascular and Metabolic Health, University of Glasgow, Manchester, GBR

**Keywords:** cardiovascular magnetic resonance imaging, diagnostic test accuracy, heart failure, n-terminal pro-b-type natriuretic peptide, personalised medicine

## Abstract

Background

Heart failure (HF) is underdiagnosed in the community. N-terminal pro-B-type natriuretic peptide (NT-proBNP) has variable sensitivity for HF across different subgroups, particularly for HF with preserved ejection fraction (HFpEF). We calculated individualised NT-proBNP thresholds with 90% sensitivity for the detection of HF, adjusted for age and comorbidities. We investigated whether individualised thresholds had greater sensitivity for the detection of HF, and whether adverse event rates were similarly low in patients with NT-proBNP levels below individualised and fixed thresholds.

Methods

A total of 1320 patients were recruited from a prospective clinical cohort referred for cardiovascular magnetic resonance (CMR). The diagnostic performance of individualised and fixed NT-proBNP thresholds for detecting HF was compared. The associations between individualised thresholds and the primary composite outcome of first HF hospitalisation or cardiovascular death were investigated.

Results

Individualised NT-proBNP thresholds ranged from 31 to 2295 pg/ml. Individualised thresholds were more sensitive for the detection of all HF (0.90; 0.80 for 125 pg/ml; 0.55 for 400 pg/ml), HFpEF (0.87; 0.74 for 125 pg/ml; 0.43 for 400 pg/ml), and HF with reduced ejection fraction (0.98; 0.94 for 125 pg/ml; 0.83 for 400 pg/ml) and achieved a high sensitivity of ~0.90 in all patient subgroups. Individualised thresholds associated with adverse outcomes (hazard ratio and 95% confidence interval 6.88 (3.04-15.60)). Patients with NT-proBNP levels below individualised thresholds and a fixed threshold of 125 pg/ml had similarly low event rates.

Conclusions

Individualised NT-proBNP thresholds, optimised for increased sensitivity, may inform future investigation in community settings.

## Introduction

N-terminal pro-B-type natriuretic peptide (NT-proBNP) plays a pivotal role in community assessment for heart failure (HF) and is a gatekeeper to echocardiography and specialist referral [1]. Despite this, HF remains significantly underdiagnosed in the community [2], and 80% of patients are diagnosed after their first HF hospitalisation [3].

In primary care, NT-proBNP thresholds of 125 pg/ml (European Society of Cardiology) [4] and 400 pg/ml (National Institute for Health and Care Excellence (NICE), UK) [5] have been shown to be sensitive (0.95 and 0.82, respectively) for HF [6,7]. However, most studies did not distinguish between HF with preserved ejection fraction(HFpEF) and HF with reduced ejection fraction (HFrEF), and NT-proBNP has lower sensitivity for HFpEF (e.g., a threshold of 125 pg/ml has a sensitivity of 0.67 for HFpEF in patients with BMI ≥ 35 kg/m^2^) [8,9].

NT-proBNP levels are significantly affected by multiple variables. Thus, the diagnostic performance of fixed NT-proBNP thresholds is likely to vary considerably with these variables [10,11]. Reflecting this, recent consensus statements advocate adjusted NT-proBNP thresholds; however, the proposed adjustments incorporate only a small number of variables (age, sex, and/or atrial fibrillation), use ordinal categorical thresholds, and require look-up tables for use [1]. A more effective solution would be a tool to calculate individualised NT-proBNP thresholds, adjusted for all relevant variables, which could be readily incorporated into electronic health records.

In this study, we aimed to develop a novel approach to calculating individualised NT-proBNP thresholds that vary according to age and key comorbidities and that retain high sensitivity for detecting HF given the current underdiagnosis. We hypothesised that compared to fixed NT-proBNP thresholds, individualised thresholds would have greater sensitivity for the detection of HF, particularly HFpEF, including across different patient subgroups. We further hypothesised that individualised NT-proBNP thresholds would remain predictive of adverse outcomes and that patients with NT-proBNP levels below the individualised thresholds would have low event rates similar to those patients with levels below fixed thresholds.

This study has been presented as an Abstract in the European Heart Journal (https://academic.oup.com/eurheartj/article/46/Supplement_1/ehaf784.1170/8310295).

## Materials and methods

Study population

**Figure 1:**
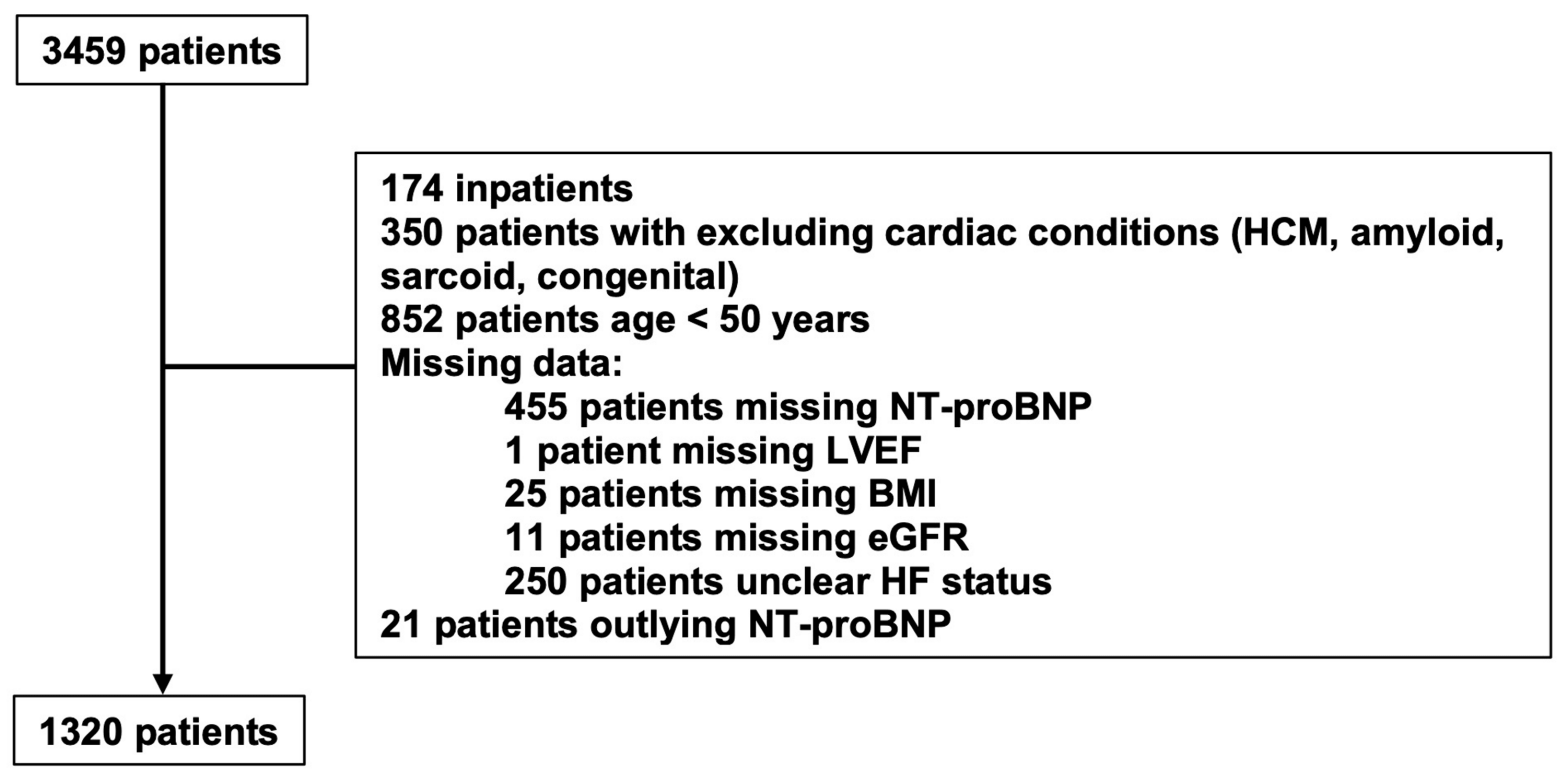
Patient flow diagram. BMI: body mass index; eGFR: estimated glomerular filtration rate; HCM: hypertrophic cardiomyopathy; HF: heart failure; LVEF: left ventricular ejection fraction; NT-proBNP: N-terminal pro-B-type natriuretic peptide.

**Figure 2:**
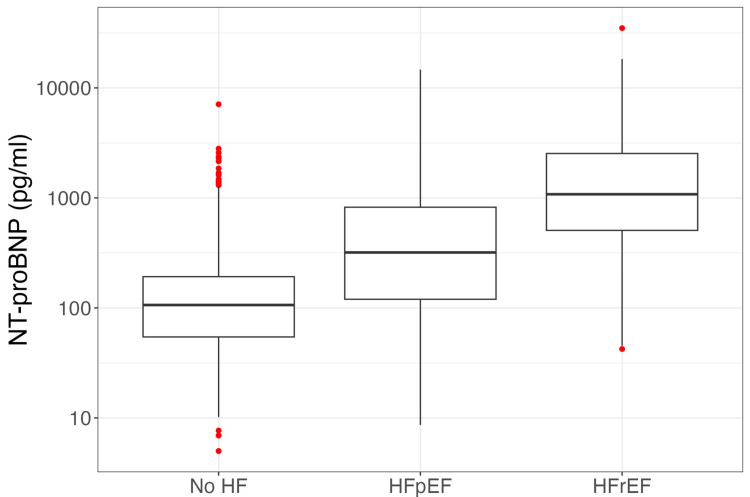
Box and whisker plot of N-terminal pro-B-type natriuretic peptide distribution between no heart failure, heart failure with preserved ejection fraction, and heart failure with reduced ejection fraction. Outliers with NT-proBNP values greater or lesser than the upper and lower quartiles plus 1.5 x interquartile range on the logarithmic scale are shown in red. HF: heart failure; HFpEF: heart failure with preserved ejection fraction; HFrEF: heart failure with reduced ejection fraction; NT-proBNP: N-terminal pro-B-type natriuretic peptide.

Between 1 June 2016 and 31 May 2018, consecutive adult patients undergoing clinically indicated cardiovascular magnetic resonance (CMR) imaging at Manchester University NHS Foundation Trust (Manchester, UK) were prospectively recruited (registered at ClinicalTrials.gov, NCT02326324). After excluding patients aged < 50 years; hospitalised patients; patients with a diagnosis of any of the following: amyloidosis, complex congenital heart disease, Fabry disease, hypertrophic cardiomyopathy, iron overload, myocarditis, and stress-induced cardiomyopathy; patients with missing data; and patients with outlier NT-proBNP values (Figures 1 and 2), patients were categorised according to HF status as follows:

1) No HF: No HF symptoms, no structural abnormalities, no history of HF, and left ventricular ejection fraction (LVEF) ≥ 50%.

2) HFpEF: HF signs and symptoms (shortness of breath, orthopnoea, or peripheral oedema), and structural abnormalities (indexed left atrial area ≥ 15 cm^2^/m^2^, left ventricle maximum wall thickness ≥ 12 mm, indexed myocardial mass ≥ 95 g/m^2^(women) or ≥ 115 g/m^2^ (men)), and LVEF > 40%.

3) HFrEF: HF signs and symptoms and LVEF ≤ 40%. No other structural abnormalities were required.

The investigation conforms with the Declaration of Helsinki. The study was approved by an NHS Research Ethics Committee (NHS North-West Greater Manchester West Research Ethics Committee 14/NW/1165), and all patients provided written informed consent.

Procedures

Data were managed using Research Electronic Data Capture (REDCap) [12]. Baseline characteristics were identified from medical records. Measurements of cardiac structure and function were made from CMR images using Circle Cardiovascular Imaging in accordance with international recommendations [13]. NT-proBNP was laboratory assessed from blood sampling performed on the same day as CMR (cobas e 411 immunoanalyser; Roche Diagnostics, Mannheim, Germany). All baseline data collection was blinded to outcome data.

Outcomes

Outcome data were obtained from NHS England. Hospital Episode Statistics for Admitted Patient Care records were used to identify episodes of hospital admission, and Hospital Episode Statistics-Office of National Statistics (civil registration) records were used to identify deaths and cause of deaths. Hospital Episode Statistics data contain diagnostic coding from the International Classification of Diseases, 10th revision (ICD-10), to denote up to 20 diagnoses per episode of hospital admission or cause of death. Hospitalisation for HF was defined as the first unscheduled admission following CMR in which HF (ICD-10 codes: I11.0, I25.5, I42.0, I42.9, I50.0, I50.1, and I50.9) was the primary diagnosis, in accordance with UK National Heart Failure Audit methodology [14]. Cardiovascular (CV) death was defined as a death due to a CV cause (ICD-10 codes: I00-I99) after CMR, and all-cause death as a death due to any cause after CMR [15]. The follow-up period was from the beginning of recruitment (1 June 2016) until 18 December 2024. NHS England provided the outcome data blinded to patient characteristics. Outcome data were available for all participants.

Statistical analysis

Continuous data are presented as mean ± (standard deviation) or median ± (interquartile range), as appropriate. Categorical data are presented as counts and percentages. Missing data were handled using complete-case analysis.

NT-proBNP was normalised using natural logarithmic transformation (ln). ln NT-proBNP, levels were compared between HF status using one-way ANOVA and Tukey pairwise testing. The relationship between NT-proBNP and baseline characteristics was assessed using univariable and multivariable linear regression models. Covariates were selected based on literature review of patient characteristics that may affect NT-proBNP levels and diagnostic performance [10,11]. Ten covariates were considered: atrial fibrillation (AF), age, body mass index (BMI), chronic obstructive pulmonary disease (COPD), current smoker, diabetes, female sex, estimated glomerular filtration rate (eGFR), hypertension, and ischaemic heart disease (IHD). Variables for which P values were <0.3 in the univariable analyses were included in the multivariable model.

To assess the diagnostic performance of NT-proBNP for the detection of HF, measures of diagnostic performance (sensitivity, specificity, positive and negative likelihood ratio, and positive and negative predictive value) were calculated. The procedure was repeated for the detection of HFpEF and HFrEF individually and in different patient subgroups categorised by covariates with significant multivariable associations with ln NT-proBNP. Receiver operating characteristic (ROC) curves were constructed using Bayesian bootstrap resampling (5000 resamples) [16].

Individualised NT-proBNP thresholds were derived using the non-parametric Bayesian inference method implemented in the ‘ROCnReg’ package [17]. In brief, this method uses a single-weights dependent Dirichlet process to model covariate-specific ROC curves that change as a function of covariates [17-19]. Five covariates (age, eGFR, BMI, AF, and IHD) were included in the model because of significant associations with ln NT-proBNP and significant effects on diagnostic performance in patient subgroups. The relationship between ln NT-proBNP and continuous covariates were modelled with cubic splines. Interaction effects were not included. The covariate-specific ROC curves were used to calculate NT-proBNP thresholds with 90% sensitivity for HF, adjusted for covariates.

Net reclassification improvement (NRI) tables were used to assess the impact of individualised versus fixed NT-proBNP thresholds [20]. The NRI was calculated for the net proportion of patients with HF correctly reclassified as HF, and the net proportion of patients without HF correctly reclassified as no HF. The NRI was tested for statistical significance as previously described [20].

To assess the prognostic significance of fixed and individualised NT-proBNP thresholds, the primary composite outcome was defined as time to first HF hospitalisation or CV death. Secondary outcomes included each component of the primary outcome individually and time to all-cause death. Kaplan-Meier plots were constructed and stratified according to fixed and individualised NT-proBNP thresholds. The hazard ratio (HR) for the primary composite outcome was determined using Fine-Gray analysis, with non-CV death included as a competing risk [21]. The HR for each component of the primary outcome was determined with all-cause death and non-CV death included as competing risks, respectively. The HR for all-cause death was determined using Cox regression analysis [22]. Schöenfeld residuals were used to evaluate the proportional hazards assumption. All statistical analyses were performed using R version 4.4 (R Foundation for Statistical Computing, Vienna, Austria).

## Results

Patient characteristics

**Figure 3:**
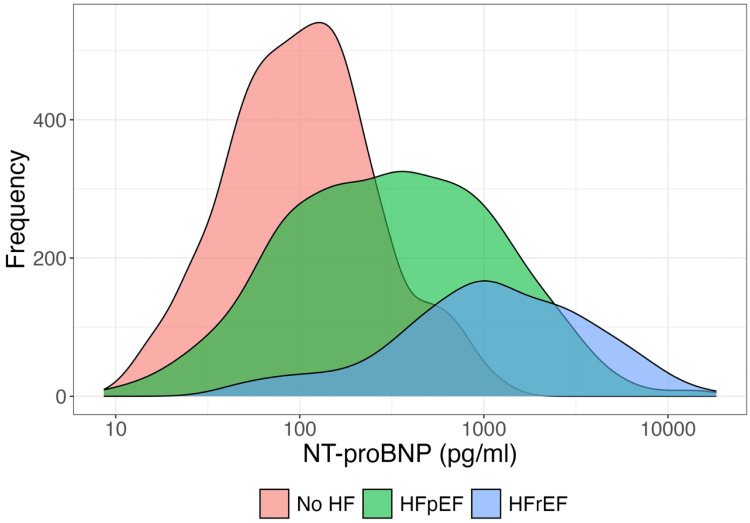
NT-proBNP distribution between patients with no HF, HFpEF, and HFrEF. HF: heart failure; HFpEF: heart failure with preserved ejection fraction; HFrEF: heart failure with reduced ejection fraction; NT-proBNP: N-terminal pro-B-type natriuretic peptide.

A total of 1320 patients were included in the study. Baseline characteristics are presented in Table 1. Around 57.1% were classified as having HF (754/1320), including HFpEF (40.6%, 536/1320) and HFrEF (16.5%, 218/1320). NT-proBNP levels were significantly higher in patients with HFrEF compared to HFpEF and those without HF (P <0.001 for one-way ANOVA and pairwise comparisons; Figure 3). CMR scanning indications are shown in Table 2.

**Table 1:** Baseline characteristics. Values are n (%) or mean ± standard deviation. BMI: body mass index; COPD: chronic obstructive pulmonary disease; ECV: extracellular volume; eGFR: estimated glomerular filtration rate; HF: heart failure; ln: natural logarithm; LVEF: LV ejection fraction; NT-proBNP: N-terminal pro-B-type natriuretic peptide.

	No HF (N = 566)	HFpEF (N = 536)	HFrEF (N = 218)	Overall (N = 1320)
Demographics and comorbidities				
Age (years)	62.6 (8.2)	66.4 (8.8)	65.0 (8.9)	64.6 (8.7)
Female sex (%)	219 (38.7%)	190 (35.4%)	56 (25.7%)	465 (35.2%)
White ethnicity (%)	492 (86.9%)	486 (90.7%)	199 (91.3%)	1177 (89.2%)
BMI (kg/m^2^)	28.5 (5.6)	29.1 (5.7)	29.0 (6.1)	28.9 (5.8)
Comorbidity				
Obesity (BMI ≥ 30 kg/m^2^) (%)	188 (33.2%)	215 (40.1%)	76 (34.9%)	479 (36.3%)
Hypertension (%)	281 (49.6%)	349 (65.1%)	132 (60.6%)	762 (57.7%)
Diabetes (%)	91 (16.1%)	98 (18.3%)	50 (22.9%)	239 (18.1%)
Atrial fibrillation (%)	56 (9.9%)	144 (26.9%)	63 (28.9%)	263 (19.9%)
Peripheral vascular disease (%)	21 (3.7%)	34 (6.3%)	16 (7.3%)	71 (5.4%)
Stroke (%)	37 (6.5%)	52 (9.7%)	18 (8.3%)	107 (8.1%)
Hyperlipidaemia (%)	311 (54.9%)	322 (60.1%)	130 (59.6%)	763 (57.8%)
Ischaemic heart disease (%)	231 (40.8%)	258 (48.1%)	109 (50.0%)	598 (45.3%)
COPD (%)	13 (2.3%)	49 (9.1%)	24 (11.0%)	86 (6.5%)
Family history of cardiovascular disease (%)	326 (57.6%)	280 (52.2%)	112 (51.4%)	718 (54.4%)
Current smoker (%)	29 (5.1%)	42 (7.8%)	32 (14.7%)	103 (7.8%)
Ex-smoker (%)	217 (38.3%)	249 (46.5%)	106 (48.6%)	572 (43.3%)
Laboratory measurements				
eGFR (ml/min/1.73 m^2^)	81.0 (70.0-90.0)	78.0 (65.0-90.0)	76.0 (60.0-89.0)	79.0 (66.0-90.0)
NT-proBNP (pg/ml)	102.9 (54.4-184.4)	319.2 (119.9-822.6)	1079.5 (509.9-2522.5)	192.2 (82.7-655.8)
ln NT-proBNP	4.6 (4.0-5.2)	5.8 (4.8-6.7)	7.0 (6.2-7.8)	5.3 (4.4-6.5)
Hs-TropT (pg/ml)	9.0 (4.6-13.4)	12.2 (7.0-19.5)	16.7 (11.1-26.5)	11.1 (5.9-17.7)
Cardiac structure and function				
LA area indexed (cm^2^/m^2^)	13.0 (2.5)	15.9 (3.9)	16.5 (4.0)	14.8 (3.7)
LV mass indexed (g/m^2^)	52.4 (13.6)	60.4 (17.1)	76.0 (23.0)	59.5 (18.7)
LV end-diastolic volume indexed (ml/m^2^)	79.0 (17.1)	87.6 (24.1)	131.7 (37.0)	91.2 (30.4)
LV end-systolic volume indexed (ml/m^2^)	30.3 (9.9)	38.3 (16.7)	92.3 (34.0)	43.8 (28.7)
LVEF (%)	62.2 (6.7)	57.5 (9.5)	31.1 (7.0)	55.2 (13.6)

**Table 2:** CMR scanning indications. The total number exceeds 1320 patients because some patients had more than one scanning indication. CMR: cardiovascular magnetic resonance.

	Number of participants (N = 1320)
Ischaemic heart disease	892 (68%)
Suspected heart failure	351 (27%)
Suspected cardiomyopathy	307 (23%)
Suspected myocarditis	65 (5%)
Aortic disease	90 (7%)
Valvular heart disease	69 (5%)
Arrhythmia	167 (13%)
Congenital heart disease	24 (2%)

Associations between NT-proBNP and baseline characteristics

Univariable associations between baseline characteristics and ln NT-proBNP are presented in Table 3. Of the ten covariates considered, only female sex was not associated with ln NT-proBNP (standardised regression coefficient and 95% CI: -0.03 (-0.16-0.10), P = 0.637) and was therefore excluded from multivariable analysis. Multivariable associations between baseline characteristics and ln NT-proBNP are presented in Table 4. After multivariable adjustment, significant associations were noted between ln NT-proBNP and AF, age, BMI, COPD, current smoking, eGFR, and IHD (Table 4).

**Table 3:** Univariable associations between NT-proBNP and patient characteristics. Standardised regression coefficients represent standard deviation change in log NT-proBNP per standard deviation change in continuous variables or categorical factor. *Continuous variables centred and scaled to 1 standard deviation. AF: atrial fibrillation; BMI: body mass index; COPD: chronic obstructive pulmonary disease; CI: confidence interval; eGFR: estimated glomerular filtration rate; IHD: ischaemic heart disease; NT-proBNP: N-terminal pro-B-type natriuretic peptide; SE: standard error.

Variable	Univariable associations				
	Intercept	Regression coefficient (SE)	95% CI	T statistic	P value
AF	-0.12	0.59 (0.07)	0.46-0.73	8.88	<0.001
Age (year)*	0.00	0.34 (0.03)	0.21-0.47	13.01	<0.001
BMI (kg/m^2^)*	0.00	-0.06 (0.03)	-0.20-0.07	-2.36	0.018
COPD	-0.03	0.41 (0.11)	0.28-0.54	3.73	<0.001
Current smoker	-0.02	0.30 (0.10)	0.17-0.43	2.96	0.003
Diabetes	-0.03	0.18 (0.07)	0.05-0.31	2.52	0.012
Female sex	0.01	-0.03 (0.06)	-0.16-0.10	-0.47	0.637
eGFR (ml/min/1.73 m^2^)*	0.00	-0.23 (0.03)	-0.36 to -0.10	-8.68	<0.001
Hypertension	-0.12	0.20 (0.06)	0.07-0.33	3.60	<0.001
IHD	-0.12	0.26 (0.05)	0.12-0.39	4.65	<0.001

**Table 4:** Multivariable associations between NT-proBNP and patient characteristics. Standardised regression coefficients represent standard deviation change in log NT-proBNP per standard deviation change in continuous variables or categorical factor. *Continuous variables centred and scaled to 1 standard deviation. AF: atrial fibrillation; BMI: body mass index; COPD: chronic obstructive pulmonary disease; CI: confidence interval; eGFR: estimated glomerular filtration rate; IHD: ischaemic heart disease; NT-proBNP: N-terminal pro-B-type natriuretic peptide; SE: standard error.

Variable	Multivariable model (Adjusted R^2^ 0.20)			
	Regression coefficient (SE)	95% CI	T statistic	P value
Intercept	-0.27 (0.05)	-0.36 to -0.19	-6.04	<0.001
AF	0.49 (0.06)	0.36-0.61	7.78	<0.001
Age (year)*	0.26 (0.03)	0.21-0.31	9.74	<0.001
BMI (kg/m^2^)*	-0.07 (0.03)	-0.12 to -0.02	-2.68	0.007
COPD	0.31 (0.10)	0.12-0.51	3.13	0.002
Current smoker	0.44 (0.09)	0.25-0.62	4.68	<0.001
Diabetes	0.12 (0.07)	-0.01-0.25	1.83	0.067
eGFR (ml/min/1.73 m^2^)*	-0.14 (0.03)	-0.19 to -0.09	-5.55	<0.001
Hypertension	0.05 (0.05)	-0.06-0.15	0.84	0.399
IHD	0.16 (0.05)	0.06-0.26	3.23	<0.001

Diagnostic performance of fixed NT-proBNP thresholds

**Figure 4:**
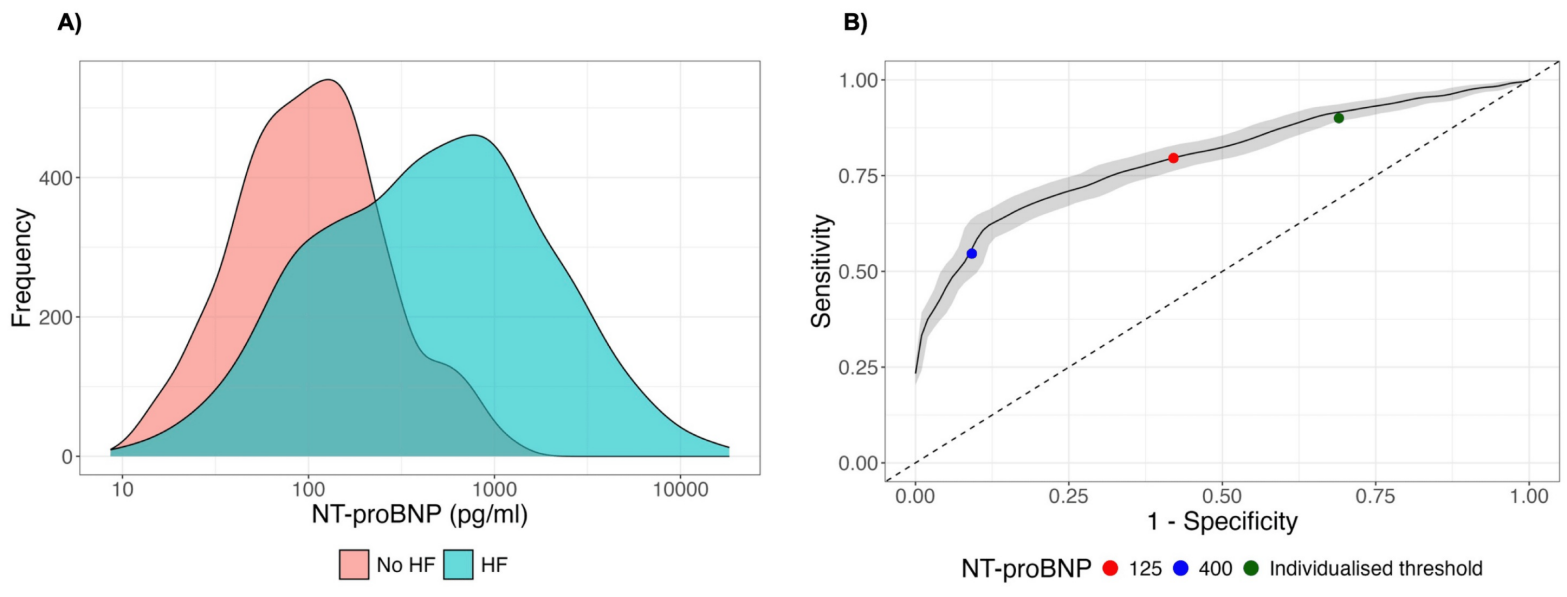
Diagnostic performance of NT-proBNP for detection of all HF subtypes. (A) Distribution of NT-proBNP between patients with and without HF. (B) ROC curve posterior mean (black line) and 95% credible interval (shaded area) for NT-proBNP thresholds for the detection of all HF. NT-proBNP thresholds of 125 pg/ml (red point), 400 pg/ml (blue point), and individualised thresholds (green point) are highlighted. HF: heart failure; NT-proBNP: N-terminal pro-B-type natriuretic peptide; ROC: receiver operating characteristic.

**Figure 5:**
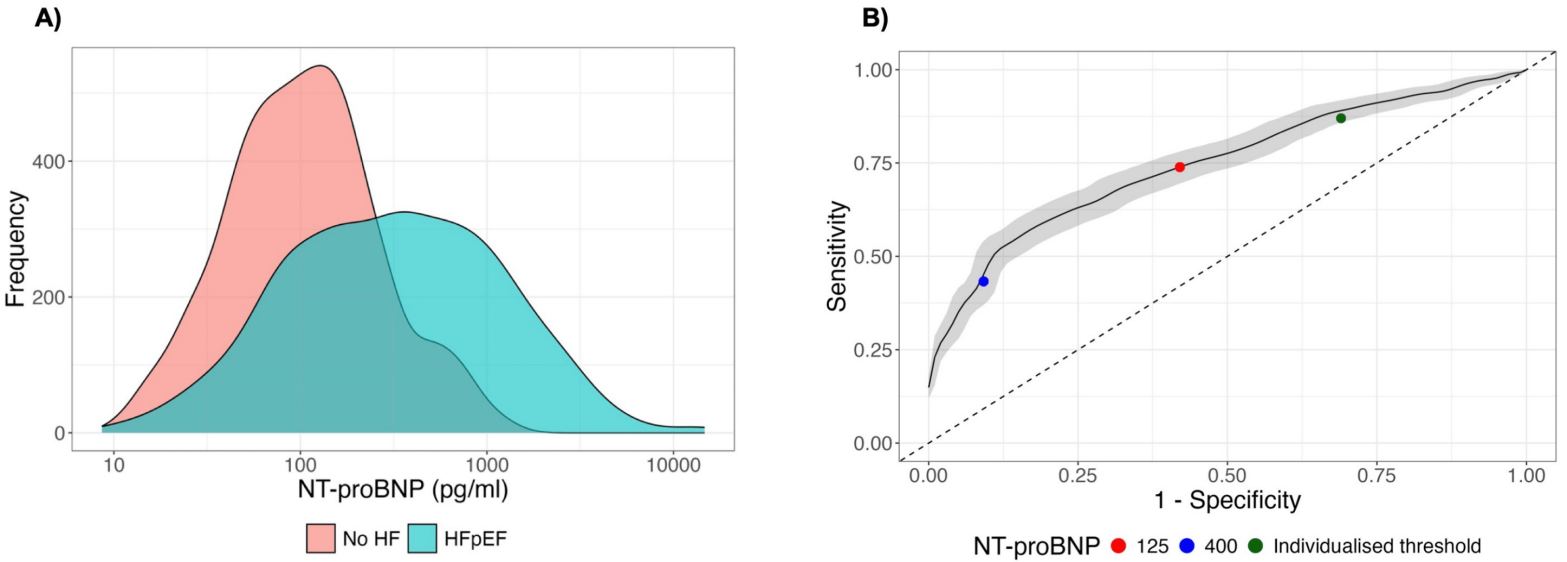
Diagnostic performance of NT-proBNP for HFpEF diagnosis. (A) Distribution of NT-proBNP between patients with and without HFpEF. (B) ROC curve posterior mean (black line) and 95% credible interval (shaded area) for use of NT-proBNP thresholds for HFpEF diagnosis. NT-proBNP thresholds of 125 pg/ml (red point), 400 pg/ml (blue point), and individualised thresholds (green point) are highlighted. Patients with HFrEF were excluded from this analysis. HF: heart failure; HFpEF: heart failure with preserved ejection fraction; HFrEF: heart failure with reduced ejection fraction; NT-proBNP: N-terminal pro-B-type natriuretic peptide.

**Figure 6:**
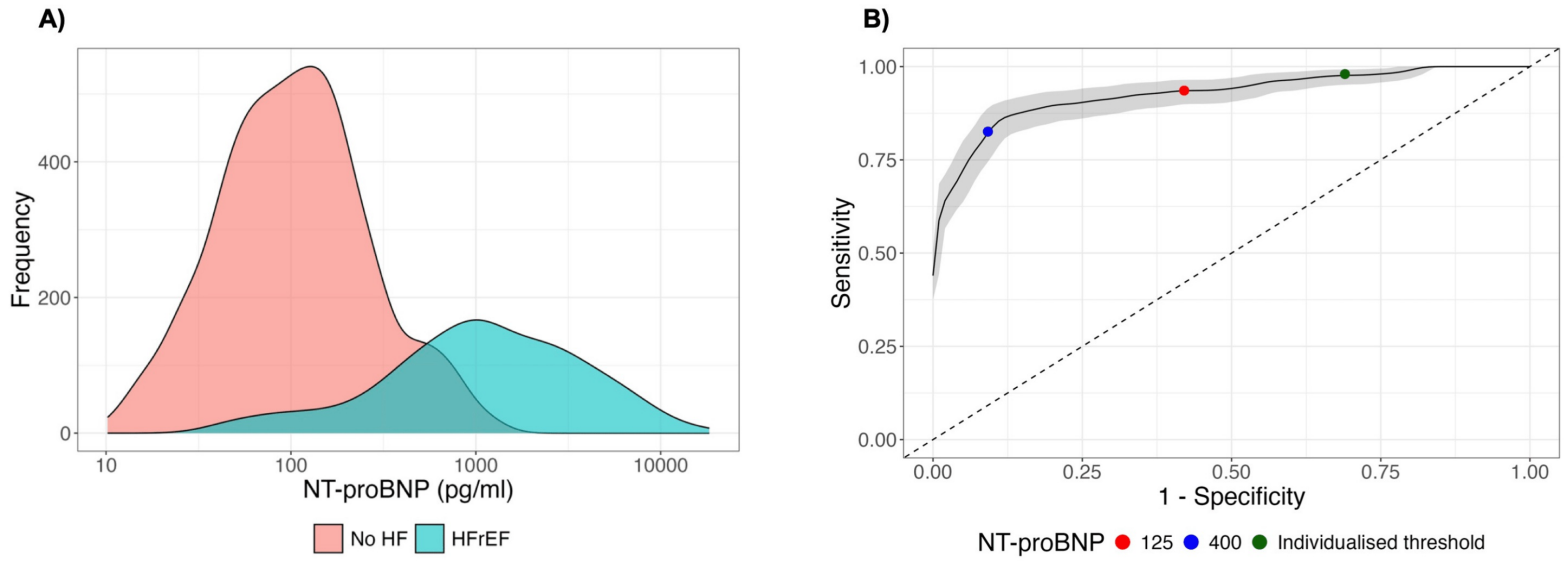
Diagnostic performance of NT-proBNP for HFrEF diagnosis. (A) Distribution of NT-proBNP between patients with and without HFrEF. (B) ROC curve posterior mean (black line) and 95% credible interval (shaded area) for use of NT-proBNP thresholds for HFrEF diagnosis. NT-proBNP thresholds of 125 pg/ml (red point), 400 pg/ml (blue point), and individualised thresholds (green point) are highlighted. Patients with HFpEF were excluded from this analysis. HF: heart failure; HFpEF: heart failure with preserved ejection fraction; HFrEF: heart failure with reduced ejection fraction; NT-proBNP: N-terminal pro-B-type natriuretic peptide.

The summary diagnostic performance of NT-proBNP is shown in Table 5. At thresholds of 125 and 400 pg/ml, NT-proBNP had limited sensitivity for detecting all HF (0.80 and 0.55, respectively; Figure 4) and HFpEF (0.74 and 0.43, respectively; Figure 5), but was highly sensitive for detecting HFrEF (sensitivity 0.94 and 0.83, respectively; Figure 6).

**Table 5:** Diagnostic performance of NT-proBNP for diagnosis of HF, HFpEF, and HFrEF. *Comparison of no HF vs. HFpEF. ∆Comparison of no HF vs. HFrEF. HF: heart failure; HFpEF: heart failure with preserved ejection fraction; HFrEF: heart failure with reduced ejection fraction; LR: likelihood ratio; NPV: negative predictive value; NT-proBNP: N-terminal pro-B-type natriuretic peptide; PPV: positive predictive value.

	125 pg/ml						400 pg/ml						Adjusted threshold					
	Sensitivity	Specificity	LR+	LR-	PPV	NPV	Sensitivity	Specificity	LR+	LR-	PPV	NPV	Sensitivity	Specificity	LR+	LR-	PPV	NPV
All HF	0.80	0.58	1.89	0.35	0.72	0.68	0.55	0.91	5.95	0.50	0.89	0.60	0.90	0.31	1.30	0.32	0.63	0.70
HFpEF*	0.74	0.58	1.76	0.45	0.62	0.70	0.43	0.91	4.71	0.62	0.82	0.63	0.87	0.31	1.26	0.43	0.54	0.71
HFrEF^∆^	0.94	0.58	2.23	0.11	0.46	0.96	0.83	0.91	8.99	0.19	0.78	0.93	0.98	0.31	1.42	0.07	0.35	0.97

At thresholds of 125 and 400 pg/ml, significant differences were noted in the diagnostic performance of NT-proBNP within patient subgroups (Table 6). The lowest sensitivity was noted in patients without atrial fibrillation (sensitivity 0.76 and 0.49, respectively), age < 75 years (sensitivity 0.77 and 0.52, respectively), BMI ≥ 30 kg/m^2^ (sensitivity 0.75 and 0.52, respectively), eGFR ≥ 60 ml/min/m^2^ (sensitivity 0.78 and 0.52, respectively), and without IHD (sensitivity 0.75 and 0.51, respectively). The NT-proBNP thresholds with 90% sensitivity for the detection of HF in these subgroups were 64, 69, 63, 69, and 58 pg/ml, respectively (Table 6). The diagnostic performance of NT-proBNP was similar irrespective of current smoking and COPD status (Table 6).

**Table 6:** NT-proBNP thresholds for diagnosis of all HF categorised by baseline characteristics. AF: atrial fibrillation; BMI: body mass index; COPD: chronic obstructive pulmonary disease; HF: heart failure; eGFR: estimated glomerular filtration rate; IHD: ischaemic heart disease; NT-proBNP: N-terminal pro-B-type natriuretic peptide.

	NT-proBNP 125 pg/ml		NT-proBNP 400 pg/ml		Individualised threshold		NT-proBNP with 90% sensitivity (pg/ml)
	Sensitivity	Specificity	Sensitivity	Specificity	Sensitivity	Specificity	
Atrial fibrillation							
No (N = 1057)	0.76	0.59	0.49	0.91	0.89	0.29	64
Yes (N = 263)	0.88	0.45	0.71	0.88	0.92	0.46	105
Age (years)							
<75 (N = 1133)	0.77	0.61	0.52	0.93	0.90	0.32	69
≥75 (N = 187)	0.91	0.27	0.69	0.70	0.90	0.21	141
BMI (kg/m^2^)							
<30 (N = 841)	0.83	0.59	0.56	0.90	0.90	0.36	76
≥30 (N = 479)	0.75	0.56	0.52	0.92	0.90	0.21	63
COPD							
No (N = 1234)	0.80	0.58	0.54	0.91	-	-	72
Yes (N = 86)	0.75	0.54	0.58	0.85	-	-	69
Current smoker							
No (N = 1217)	0.80	0.58	0.54	0.91	-	-	70
Yes (N = 103)	0.80	0.52	0.58	0.86	-	-	84
eGFR (ml/min/m^2^)							
<45 (N = 28)	1.00	0.00	0.76	0.71	0.90	0.29	312
45-59 (N = 130)	0.85	0.34	0.66	0.71	0.88	0.40	81
≥60 (N = 1162)	0.78	0.60	0.52	0.92	0.90	0.31	69
IHD							
No (N = 722)	0.75	0.64	0.51	0.94	0.88	0.38	58
Yes (N = 598)	0.84	0.50	0.59	0.86	0.92	0.22	83

Diagnostic performance of individualised NT-proBNP thresholds

Individualised NT-proBNP thresholds were derived after adjusting for age, eGFR, BMI, AF, and IHD. The other covariates were not used to derive the thresholds because of non-significant associations with ln NT-proBNP (sex, diabetes, and hypertension) or minimal effect on diagnostic performance (current smoking and COPD).

**Figure 7:**
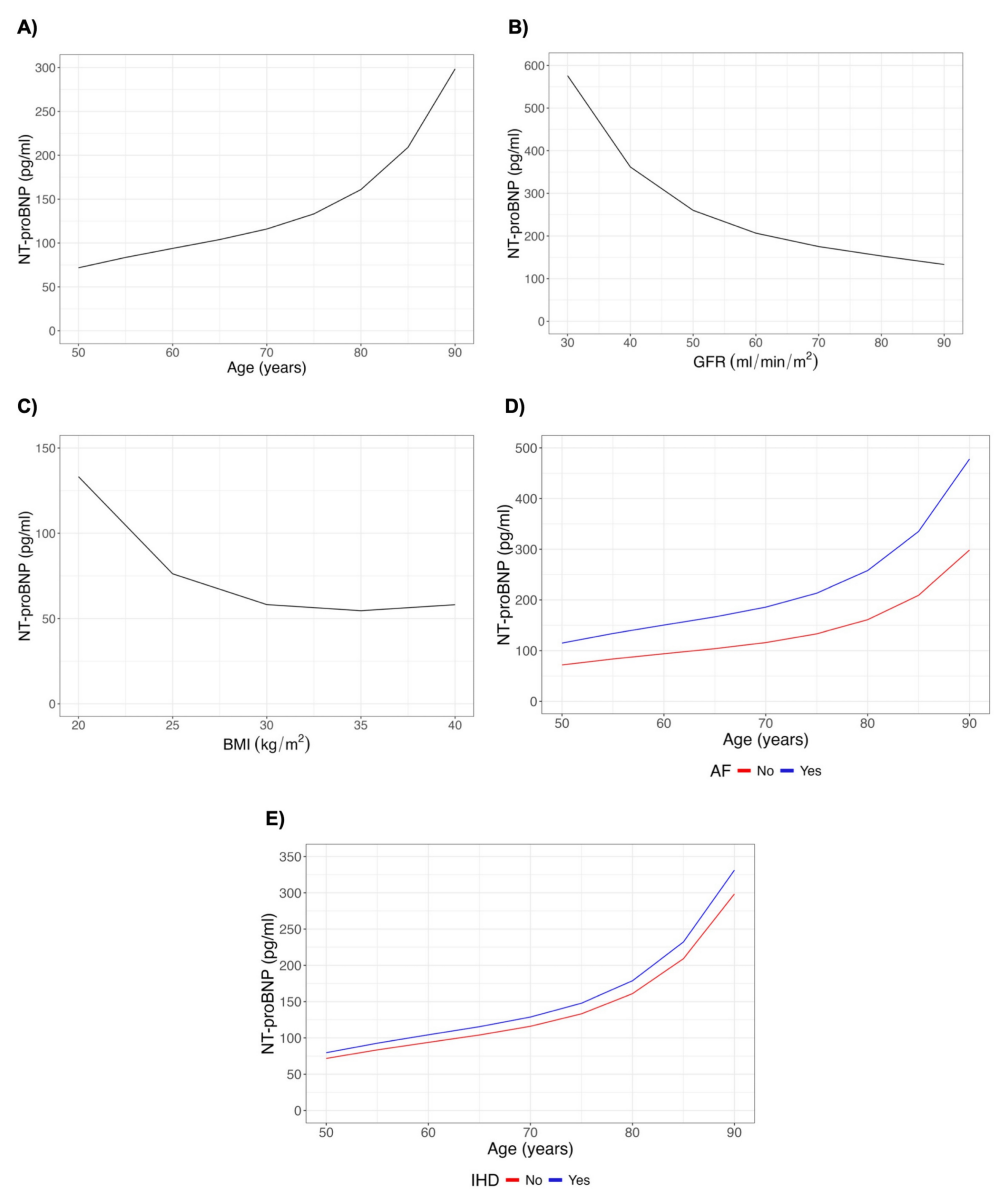
NT-proBNP thresholds with 90% sensitivity for all HF diagnoses. (A) Thresholds by age, adjusted for eGFR 90 ml/min/m^2^, BMI 20 kg/m^2^, no AF, and no IHD. (B) Thresholds by GFR, adjusted for age 75 years, BMI 20 kg/m^2^, no AF, and no IHD. (C) Thresholds by BMI, adjusted for age 75 years, eGFR 90 ml/min/m^2^, no AF, and no IHD. (D) Thresholds by age and AF, adjusted for eGFR 90 ml/min/m^2^, BMI 20 kg/m^2^, and no IHD. (E) Thresholds by age and IHD, adjusted for eGFR 90 ml/min/m^2^, BMI 20 kg/m^2^, and no AF. AF: atrial fibrillation; BMI: body mass index; eGFR: estimated glomerular filtration rate; IHD: ischaemic heart disease; NT-proBNP: N-terminal pro-B-type natriuretic peptide.

Individualised NT-proBNP thresholds with 90% sensitivity for all HF ranged from 31 pg/ml (for a hypothetical patient with age 50 years, eGFR 90 ml/min/m^2^, BMI 40 kg/m^2^, no AF, and no IHD) to 2295 pg/ml (for a hypothetical patient with age 90 years, eGFR 30 ml/min/m^2^, BMI 20 kg/m^2^, AF, and IHD) (Figure 7). The summary diagnostic performance of individualised NT-proBNP thresholds are shown in Table 5. Compared to 125 and 400 pg/ml, individualised NT-proBNP thresholds resulted in a higher sensitivity for the detection of all HF (sensitivity 0.90 for individualised threshold, 0.80 for 125 pg/ml, and 0.55 for 400 pg/ml), HFpEF (sensitivity 0.87 for individualised threshold, 0.74 for 125 pg/ml, and 0.43 for 400 pg/ml), and HFrEF (sensitivity 0.98 for individualised threshold, 0.94 for 125 pg/ml, and 0.83 for 400 pg/ml). Individualised NT-proBNP thresholds resulted in a sensitivity of ~0.90 for the detection of HF in all subgroups (Table 6).

**Figure 8:**
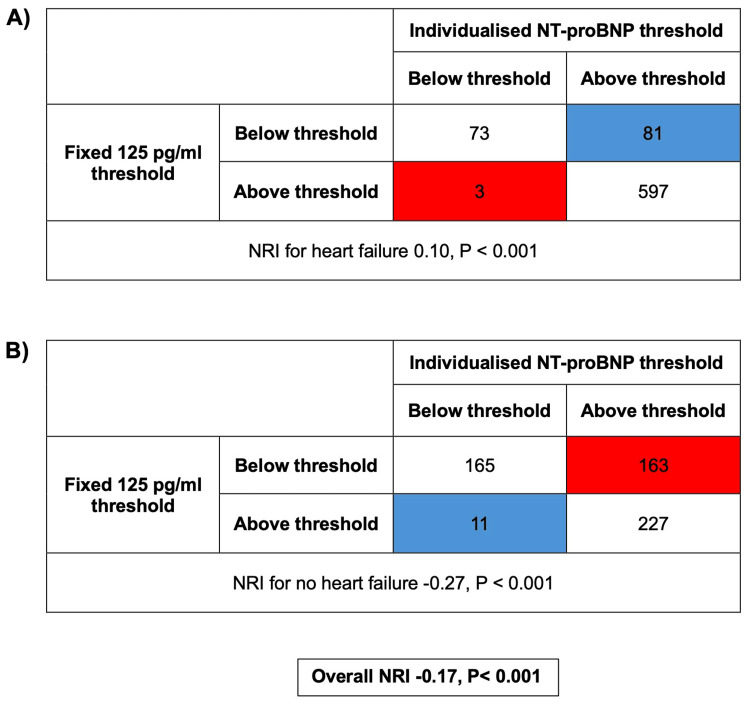
Net reclassification index tables for individualised NT-proBNP thresholds vs. fixed threshold of 125 pg/ml. (A) NRI table for patients with heart failure. (B) NRI table for patients without heart failure. Blue box correctly reclassified, and red box incorrectly reclassified. NRI: net reclassification index; NT-proBNP: N-terminal pro-B-type natriuretic peptide.

Compared to a 125 pg/ml threshold, individualised NT-proBNP thresholds resulted in the detection of an additional 78 HF cases (69 HFpEF and 9 HFrEF; 13.0% relative increase) at the cost of an additional 230 positive tests (27.4% relative increase), i.e. 1 additional HF case for every 2.9 additional positive tests. Compared to a 125 pg/ml threshold, individualised NT-proBNP thresholds improved the reclassification of patients with HF (NRI 0.10, P < 0.001, Figure 8) at the cost of worsening the reclassification of patients without HF (NRI -0.27, P < 0.001, Figure 8).

**Figure 9:**
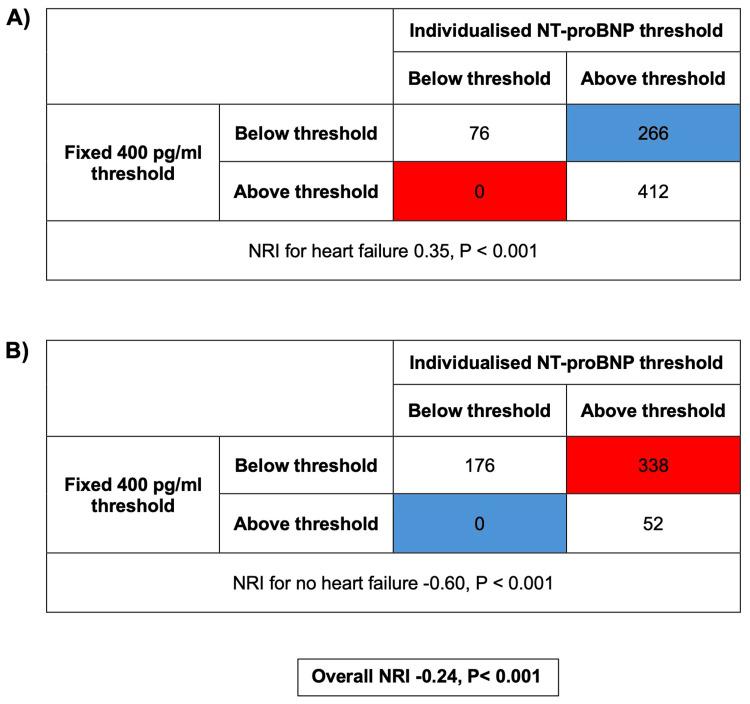
Net reclassification index tables for individualised NT-proBNP thresholds vs. fixed threshold of 400 pg/ml. (A) NRI table for patients with heart failure. (B) NRI table for patients without heart failure. Blue box correctly reclassified, and red box incorrectly reclassified. NRI: net reclassification index; NT-proBNP: N-terminal pro-B-type natriuretic peptide.

Compared to a 400 pg/ml threshold, individualised NT-proBNP thresholds resulted in the detection of an additional 266 HF cases (233 HFpEF and 33 HFrEF; 64.6% relative increase) at the cost of an additional 604 positive tests (130.2% relative increase), i.e. 1 additional HF case for every 2.5 additional positive tests. Compared to a 400 pg/ml threshold, individualised NT-proBNP thresholds improved the reclassification of patients with HF (NRI 0.35, P < 0.001, Figure 9) at the cost of worsening the reclassification of patients without HF (NRI -0.60, P < 0.001, Figure 9).

Prognostic significance of NT-proBNP thresholds

Median follow-up duration was 2620 days (IQR 2433-2784). The primary composite outcome occurred in 168 (12.7%) patients. Individually, first HF hospitalisation occurred in 103 patients (7.8%), CV death in 96 patients (7.3%), and all-cause death in 251 patients (19.0%).

**Figure 10:**
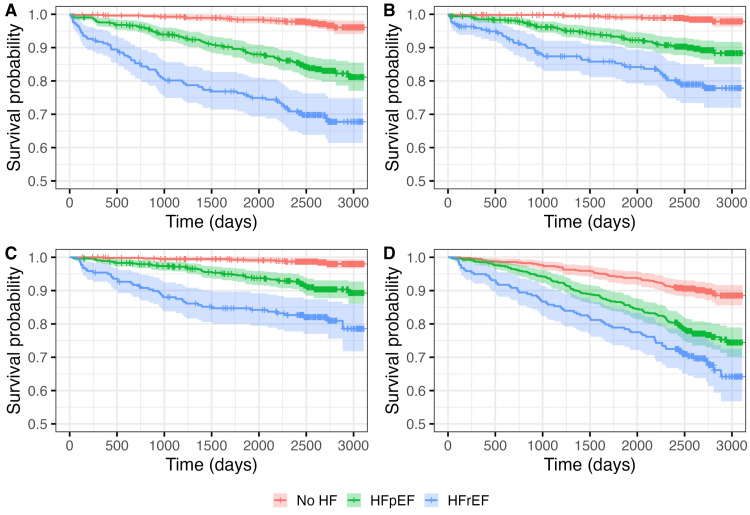
Kaplan-Meier plots for adverse outcomes in patients with no HF, HFpEF, and HFrEF. (A) Time to first HF hospitalisation or CV death. (B) Time to first HF hospitalisation. (C) Time to CV death. (D) Time to all-cause death. HF: heart failure; HFpEF: heart failure with preserved ejection fraction; HFrEF: heart failure with reduced ejection fraction; CV: cardivascular.

Significant associations were noted between HF status and adverse outcomes, with higher event rates in HFrEF compared to those with HFpEF or without HF (log rank test P < 0.001, Figure 10).

**Figure 11:**
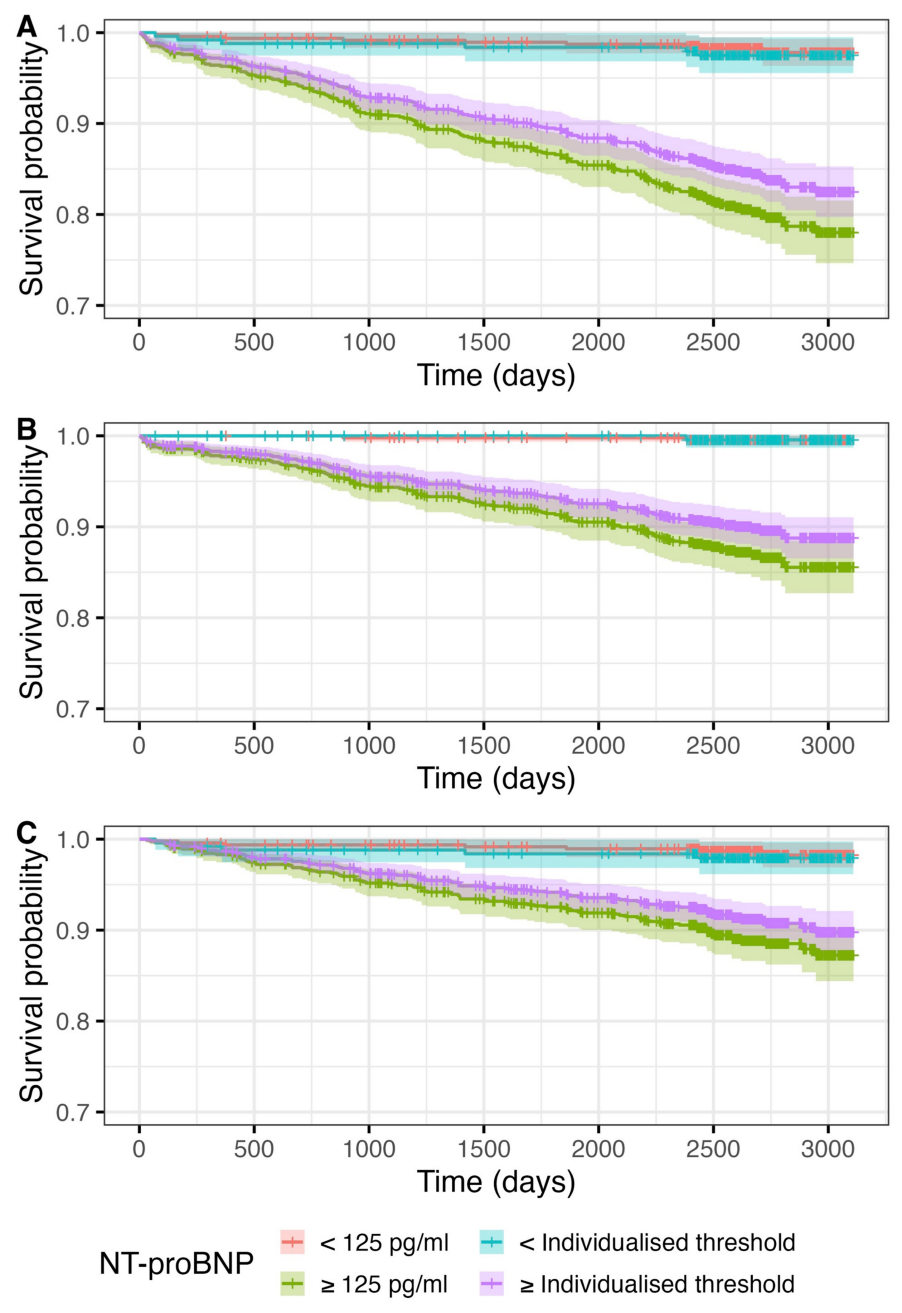
Comparison of Kaplan-Meier plots for adverse outcomes between NT-proBNP 125 pg/ml and individualised thresholds. (A) Time to first HF hospitalisation or CV death. (B) Time to first HF hospitalisation. (C) Time to CV death. NT-proBNP: N-terminal pro-B-type natriuretic peptide; HF: heart failure; CV: cardivascular.

**Figure 12:**
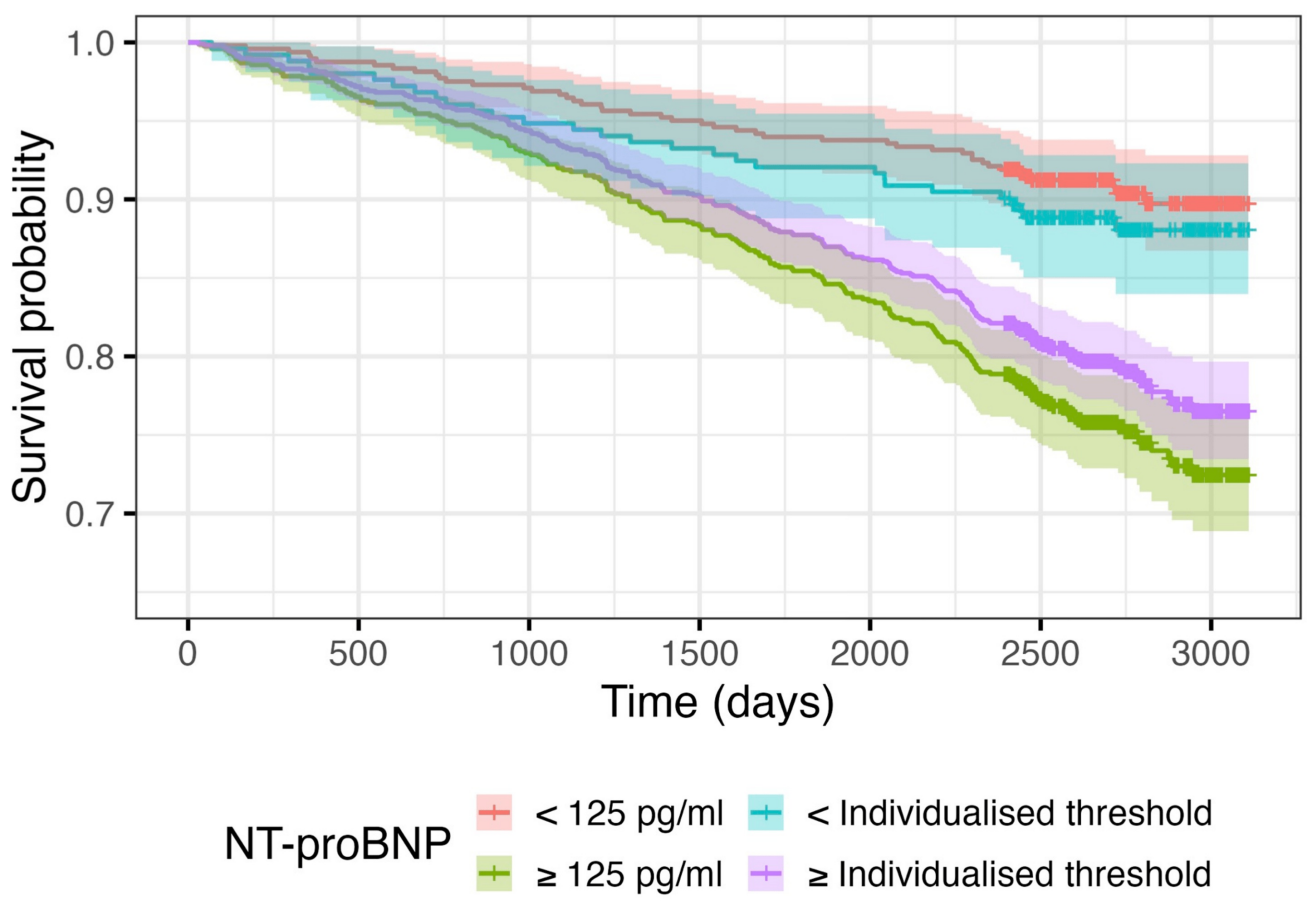
Kaplan-Meier plot for time to all-cause death between NT-proBNP 125 pg/ml and individualised thresholds. NT-proBNP: N-terminal pro-B-type natriuretic peptide.

**Figure 13:**
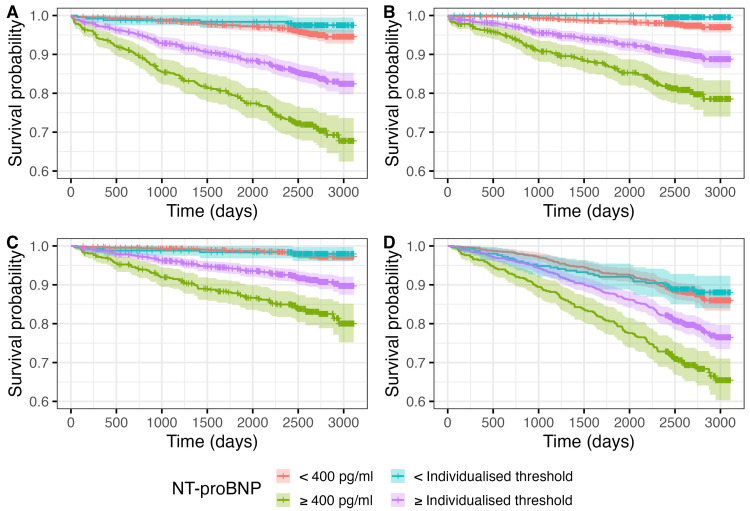
Comparison of Kaplan-Meier plots for adverse outcomes between NT-proBNP 400 pg/ml and individualised thresholds. (A) Time to first HF hospitalisation or CV death. (B) Time to first HF hospitalisation. (C) Time to CV death. (D) Time to all-cause death. NT-proBNP: N-terminal pro-B-type natriuretic peptide; HF: heart failure; CV: cardiovascular.

Kaplan-Meier plots stratified by NT-proBNP levels above and below fixed and individualised thresholds are shown in Figures 11-13. Fixed NT-proBNP thresholds were prognostically significant, with higher event rates noted in patients with NT-proBNP levels above 125 pg/ml (HR and 95% CI for primary outcome 11.20 (5.75-22.00), Table 7) and 400 pg/ml (HR and 95% CI for primary outcome 7.13 (4.99-10.20), Table 7). Individualised NT-proBNP thresholds were also prognostically significant, with significantly higher event rates in patients with NT-proBNP levels above their individualised threshold (HR and 95% CI for primary outcome 6.88 (3.04-15.60), Table 7). HR for secondary outcomes are shown in Table 8. Low event rates were noted in patients with NT-proBNP levels less than their individualised threshold, comparable to the events rates seen for patients with NT-proBNP less than 125 pg/ml (Figure 11).

**Table 7:** Prognostic significance of NT-proBNP thresholds for primary outcome of HF hospitalisation or CV death. CI: confidence interval; CV: cardiovascular; HF: heart failure; HR: hazard ratio; NT-proBNP: N-terminal pro-B-type natriuretic peptide.

	HF hospitalisation or CV death			
	HR	95% CI	Chi-square statistic	P value
125 pg/ml	11.20	5.75-22.00	49.98	<0.001
400 pg/ml	7.13	4.99-10.20	116.64	<0.001
Individualised threshold	6.88	3.04-15.60	21.44	<0.001

**Table 8:** Prognostic significance of NT-proBNP thresholds for secondary outcomes of first HF hospitalisation, CV death, and all-cause death. CI: confidence interval; HF: heart failure; HR: hazard ratio; NTproBNP: N-terminal pro-B-type natriuretic peptide; CV: cardiovascular.

	HF hospitalisation				CV death					All-cause death				
	HR	95% CI	Chi-square statistic	P value	HR		95% CI	Chi-square statistic	P value	HR		95% CI	Chi-square statistic	P value
125 pg/ml	31.1	7.67-126.0	23.23	<0.001	7.72		3.58-16.70	27.14	<0.001	2.89		2.09-3.99	41.47	<0.001
400 pg/ml	7.92	4.91-12.80	72.25	<0.001	7.65		4.68-12.50	66.1	<0.001	2.83		2.20-3.63	66.28	<0.001
Individualised threshold	25.5	3.57-182.00	10.43	<0.001	4.48		1.82-11.10	10.56	<0.001	1.91		1.30-2.81	10.73	<0.001

## Discussion

In this work, we developed a novel approach to calculate individualised NT-proBNP thresholds adjusted for age and comorbidities. We demonstrated that, compared to fixed NT-proBNP thresholds of 125 and 400 pg/ml, individualised thresholds achieved a greater sensitivity for the detection of HF, particularly HFpEF, both in the whole cohort and across patient subgroups. Finally, we demonstrated that individualised NT-proBNP thresholds remained predictive of adverse outcomes and that patients with NT-proBNP levels below their individualised thresholds had similarly low event rates to those with levels less than 125 pg/ml.

HF remains underdiagnosed in the community, and current guidelines emphasise the role of NT-proBNP as a high-sensitivity ‘rule out’ screening test [4,5]. Whilst NT-proBNP thresholds of 125 and 400 pg/ml are reported to be highly sensitive for HF in the community (0.95 and 0.82, respectively) [6,7], these studies pre-date the increased recognition of HFpEF as a clinical entity and do not differentiate diagnostic performance between HFpEF and HFrEF [6,7]. In our cohort, NT-proBNP thresholds of 125 pg/ml and 400 pg/ml were less sensitive for HFpEF compared to HFrEF, in keeping with the findings of previous studies [8,9]. As such, clinical pathways based on fixed NT-proBNP thresholds risk exacerbating the underdiagnosis of HF, particularly HFpEF.

Moreover, NT-proBNP levels are significantly affected by age and many comorbidities [10,11]. Hence, the summary diagnostic performance of fixed thresholds, without regard to their performance in patient subgroups, may be misleading for many individuals [19]. After multivariable adjustment, increasing NT-proBNP levels were associated with AF, COPD, current smoking, IHD, increasing age, and decreasing BMI and eGFR. Significant differences were noted in the diagnostic performance of NT-proBNP thresholds across patient subgroups, with the lowest sensitivity noted in patients without AF and IHD, age < 75 years, BMI ≥ 30 kg/m^2^, and eGFR ≥ 60 ml/min/m^2^.

Recent consensus statements have proposed variable NT-proBNP thresholds, adjusted for age, sex, and/or AF [1,23]. The resulting look-up tables are cumbersome to use in clinical practice and have several important limitations; they can only adjust for a small number of relevant variables, require categorisation of continuous characteristics, and cannot accommodate non-linear associations between covariates and NT-proBNP. The resulting thresholds are therefore simplified weighted averages and are unlikely to be representative of any specific patient. Individualised NT-proBNP thresholds, adjusted for all relevant variables and incorporated into electronic health records, represent a promising alternative approach for the future.

We constructed individualised NT-proBNP thresholds using a novel non-parametric Bayesian inference method, implemented within the ‘ROCnReg’ package in R [17,19]. A similar approach has been used previously in the context of D-dimer testing for pulmonary embolism [19]. This method can be used to construct diagnostic thresholds with any desired sensitivity or specificity; we selected 90% sensitivity given the current underdiagnosis of HF. Non-linear associations between continuous variables and NT-proBNP were modelled with cubic splines. We adjusted for five important clinical covariates (age, eGFR, BMI, AF, and IHD), which had significant associations with NT-proBNP and impacted on diagnostic performance. Individualised NT-proBNP thresholds ranged from 31 to 2295 pg/ml, depending on patient characteristics.

Compared to fixed NT-proBNP thresholds, individualised NT-proBNP thresholds had a greater sensitivity for the detection of all HF, HFpEF, and HFrEF, but particularly HFpEF, both for the whole cohort and across patient subgroups. The overall sensitivity and specificity for individualised NT-proBNP thresholds was 0.90 and 0.31, respectively.

In addition to its diagnostic utility, NT-proBNP is valuable as a prognostic biomarker [24]. Our results match those of previous studies, which demonstrate significant associations between NT-proBNP and adverse outcomes. Under current guidance, patients with NT-proBNP levels above 125 or 400 pg/ml are identified as high risk and warrant further investigation [4,5]. Importantly, we demonstrated that patients with NT-proBNP levels below their individualised thresholds had low event rates, comparable to the events rates seen for patients with NT-proBNP less than 125 pg/ml. This suggests that deferral of investigations may be safe in these patients, even if individualised NT-proBNP levels are above established fixed thresholds.

It is important to note that cost-effectiveness modelling was a factor in determining fixed NT-proBNP thresholds of 125 and 400 pg/ml [4,5]. The models made the explicit assumption that early diagnosis of HFpEF was less cost effective than early diagnosis of HFrEF because of a lack of clinically effective treatments for HFpEF [5]. This led to higher NT-proBNP threshold recommendations, which are targeted towards the detection of HFrEF rather than HFpEF. Such modelling needs to be revisited now that several effective treatments for HFpEF are available, including sodium-glucose cotransporter 2 inhibitors [25,26], glucagon-like 1 peptide receptor agonists [27,28], and mineralocorticoid receptor antagonists [29]. Although formal cost-effectiveness analysis was beyond the scope of this manuscript, individualised thresholds resulted in a significant increase in the number of HF cases detected with a favourable ratio relative to additional positive test results.

This study has several important limitations. The patients were recruited as part of a prospective clinical cohort referred for CMR. The characteristics of these patients will therefore differ compared to undifferentiated patients presenting to primary care with potential signs and symptoms of HF. This study only included stable outpatients, and the NT-proBNP thresholds are therefore not applicable to patients with acute decompensated HF. Several variables were not included in the NTproBNP adjustment, such as ethnicity and sex, which have previously been shown to have effects on NT-proBNP levels at a population level. Missing data were handled by complete case analysis because there is no clear framework for applying non-parametric Bayesian inference methods to multiply imputed datasets. In view of the relatively small sample size and methodological complexity, the study did not include internal validation. HF was diagnosed based on patient-reported symptoms and CMR imaging evidence of structural abnormalities and LVEF. Whilst this had the advantage of ensuring HF diagnosis was blinded to NT-proBNP, the findings require external validation in a cohort with clinically adjudicated HF. In addition, it is likely that individualised thresholds, with high sensitivity and low specificity, would result in a greater proportion of false positive test results in lower prevalence settings. The impact on downstream echocardiography and cardiology referrals requires careful assessment.

## Conclusions

In conclusion, we developed individualised NT-proBNP thresholds with 90% sensitivity for the diagnosis of HF, adjusted for age and comorbidities. We showed that individualised thresholds had greater sensitivity for the detection of HF, particularly HFpEF, compared to fixed NT-proBNP thresholds of 125 and 400 pg/ml. Finally, we showed that individualised NT-proBNP thresholds were prognostically significant, and patients with NT-proBNP levels below their individualised thresholds had similarly low event rates to those with levels less than 125 pg/ml. These findings represent a derivation-only proof of concept; external validation in an independent community cohort and assessment of decision-curve net benefit, cost-effectiveness, and implementation feasibility are required before any consideration of primary-care deployment.
